# Post-abortion contraceptive uptake, choices, and factors associated with it among women seeking abortion services in Africa: a systematic review and meta-analysis

**DOI:** 10.3389/fgwh.2025.1478797

**Published:** 2025-06-16

**Authors:** Gizachew Worku Dagnew, Melash Belachew Asresie

**Affiliations:** Department of Reproductive Health, School of Public Health, College of Medicine and Health Science, Bahir Dar University, Bahir Dar, Ethiopia

**Keywords:** post-abortion, contraceptive, family planning (FP), abortion, systematic review & metaanalysis, Africa

## Abstract

**Background:**

In Africa, over one-third of women experience multiple abortions, often due to inadequate access to effective modern contraceptives. This highlights a critical gap in understanding the patterns and predictors of post-abortion contraceptive (PAC) use. To address this issue, a systematic review and meta-analysis were conducted to assess the uptake of PAC and associated factors among African women who received abortion services.

**Methods:**

Following the PRISMA guideline, all articles published between January 1, 2015, and December 30, 2023, were systematically retrieved from multiple databases. Articles reporting PAC uptake among African women were included. The pooled prevalence of Post-abortion contraceptive uptake was determined using a random effects model. The variation between the included studies was assessed using a funnel plot and *I*^2^ heterogeneity statistics. Sources of heterogeneity: Subgroup analysis was performed by country, publication period, study design, and sub-African region.

**Results:**

From 48 articles, a total of 84,205 women who underwent abortion services were included in the analysis. The pooled prevalence of PAC uptake in Africa was 58.78% (95% CI: 52.36–65.21), with high heterogeneity (*I*^2^ = 99.8%, 95% CI: 99.2%-99.9%; *P* < 0.001). The subgroup analysis revealed variation by country, publication period, and sub-African region. The most widely used contraceptive methods were injectables (30.27%), followed by implants (25.13%), oral contraceptive pills (22.34%), and IUDs (10.47%). Attending formal education (OR = 1.46, 95% CI = 1.03, 2.07), knowing the period of fertility (OR = 1.72, 95% CI = 1.14, 2.59), counseling about contraceptives (OR = 3.40, 95% CI = 1.82, 6.35), not having a desire for pregnancy (OR = 3.08, 95% CI = 1.74, 5.35, 95% CI = 2.55, 7.42), and possessing contraceptive knowledge (OR =  2.30, 95% CI = 1.41, 3.76) had a statistically significant combined effect on PAC uptake in Africa.

**Conclusion:**

The uptake of PAC in Africa stands at 58.78%, which is considered low according to the World Health Organization's recommendation that all women should postpone conception for six months following an abortion. There was also a decline of 20.22% between 2020 and 2023 compared to the pooled uptake between 2015 and 2019. To address this, it is crucial to enhance women's awareness of post-abortion contraception, the conception risks after abortion, and to strengthen client-centered counseling alongside women's education.

**Systematic Review Registration:**

https://www.crd.york.ac.uk/PROSPERO/view/CRD42024505129, PROSPERO CRD42024505129.

## Introduction

Maternal mortality and morbidity remain significant global challenges, despite ongoing efforts to meet Sustainable Development Goal (SDG) target 3.1, which aims to reduce the global maternal mortality ratio to below 70 per 100,000 live births by 2030 (1). In 2020, an estimated 287,000 maternal deaths occurred worldwide. Ninety-five percent of maternal deaths occurred in low- and low-middle-income countries, and more than two-thirds (70%) occurred in sub-Saharan Africa (SSA) (2). Pregnancies ended with unsafe abortion can lead to maternal mortality and morbidity (3). Unsafe abortion is one of the leading causes of maternal mortality worldwide, accounting for over 13% of maternal deaths, which translates to more than 38,900 lives lost annually (4, 5).

Globally, approximately half of all pregnancies are unintended, which results in induced abortion (6); from 2015 to 2019, an estimated 121 million unintended pregnancies and 73.3 million abortions were reported each year (4). Approximately 60% of unintended pregnancies and 30% of all pregnancies result in induced abortion. Furthermore, approximately 45% of all abortions are considered unsafe, with 97% of these unsafe procedures occurring in developing countries and marginalized populations (4, 7, 8). Family planning is an essential maternal and child health service package that has the potential to reduce maternal deaths by 32%, child deaths by 10%, and unwanted pregnancies by 71% (9).

Family planning significantly benefits the health of women, children, and the community at large (10, 11). As a result of modern contraceptives, from 2019 to 2020, a total of 121 million unintended pregnancies, 21 million unsafe abortions, and 125 thousand maternal deaths were averted (12). Failing to use family planning causes unintended pregnancies that result in abortion and abortion-related complications, and death (4).

The provision of post-abortion family planning (PAFP) is one of the essential integrated postabortion care services that significantly contribute to reducing unintended pregnancies, further repeated abortions, and maternal deaths (13). The WHO recommends postponing conception for at least six months after termination of pregnancy, and all women are expected to receive post-abortion contraceptives before leaving health facilities (7). However, research evidence indicates that a significant number of women who received abortion services left the health facility without using any contraceptives, 34.2% in Ethiopia (14), 46% in Kenya (15), and 80% in Tanzania (16). Routine family planning services, such as the PAFP, should be provided for all women regardless of their socio-economic status (9, 17–19).

The WHO (7) and various national abortion guidelines (20–22) have set forth recommendations advocating for the provision of family planning services prior to discharge from healthcare facilities. These recommendations are grounded in the obligation of states to uphold sexual and reproductive health rights, which aim to postpone conception for six months and/or mitigate unintended pregnancies that may lead to repeat abortions (7, 23). Despite various efforts, a significant number of women leave these institutions without accessing essential post-abortion care services. Empirical evidence shows that between 20% and 37% of women undergo subsequent abortions, which can be attributed to the insufficient use of effective modern contraceptive methods following their initial procedure (24–26).

Although there is evidence regarding PAFP uptake at the country (27–29) and sub-continent-level (30, 31) in Africa, there is a notable lack of studies assessing its predictors. Furthermore, up-to-date data are critical for implementing intervention measures to reduce unmet family planning needs. A comprehensive understanding of the prevalence and determinants of PAFP at the continental level can foster a collaborative understanding among international stakeholders and stimulate advocacy for actions. Consequently, this systematic review and meta-analysis sought to evaluate the pooled prevalence of post-abortion contraceptive uptake and the associated predictors among women who have received abortion services in Africa. The insights derived from this research could be instrumental in developing context-specific, women-centered PAFP services for African women. Additionally, the investigation into the unintended pregnancies and the prevalence of abortions reveals that the findings could be substantially mitigate maternal morbidity and mortality within the African continent.

## Methods and materials

### Study protocol and eligibility

This study aimed to determine the pooled prevalence of post-abortion contraceptive (PAC) uptake and its determinants among women seeking abortion services in Africa through a systematic review and meta-analysis of both published and unpublished studies were performed.

The review included all cross-sectional and cohort studies conducted in various regions of Africa, focusing on the uptake and utilization of post-abortion contraceptives, reported in studies published in English between January 1, 2015, and December 30, 2023. The year 2015 was deliberately chosen as the starting point for the review since it marked the beginning of the implementation of the Sustainable Development Goals (1). The decision to exclude the older studies aimed to focus on the most relevant and current data that accurately reflects contemporary practices and trends, in line with the Sustainable Development Goals. Furthermore, the review excluded articles that had inconsistent outcome measures and/or lacked sufficient outcomes. The review and meta-analysis adhered to the guidelines outlined in the Preferred Reporting Items for Systematic Review and Meta-analysis (PRISMA) reporting checklist (32).

### Sources of information and search strategies

We searched several databases, including PubMed, Medline, Researchgate, Africa Journal Online, and Europe PMC, to retrieve relevant findings on post-abortion contraceptive uptake. We used the following mesh terms (“Abortion, Induced”), AND (“Contraceptive Agents”, OR “Family Planning Services”), AND (“Africa”), as well as a combination with specific African countries (Supplementary File S1). Additionally, we utilized Google Scholar to find unpublished articles and materials from the institutional repositories. All the search results were compiled using EndNote citation management software. Articles were last searched on January 01, 2024.

### Study selection, data extraction, and quality assurance

Using the EndNote X8 citation manager, duplicated articles were removed. Initially, all the retrieved articles were evaluated against the eligibility criteria by carefully reading the articles’ titles and abstracts. For an article to be considered eligible, its title and abstract had to include the outcome of interest: post-abortion contraceptive uptake, along with subjects involved, which were women who received abortion services, and the study setting, which needed to be in Africa. Articles that met these inclusion criteria based solely on their title or abstract were selected for full document review. Two investigators independently assessed the eligibility of each study. If any discrepancies arose between the two investigators, we discussed the differences and reviewed the full text jointly to reach a consensus. Factors considered during a consensus discussion were alignment with the research question, study design, population, outcomes, and the clarity of reporting. In our evaluation of unpublished literature, we considered several key factors: using a scientific way to determine sample size, the appropriateness of participant selection, the validity and reliability of the survey tool, the mechanisms in place to control for confounding factors and bias, and the ethical acceptability of the studies. These considerations were particularly important given that these articles did not provide evidence of peer review.

**Figure 1:**
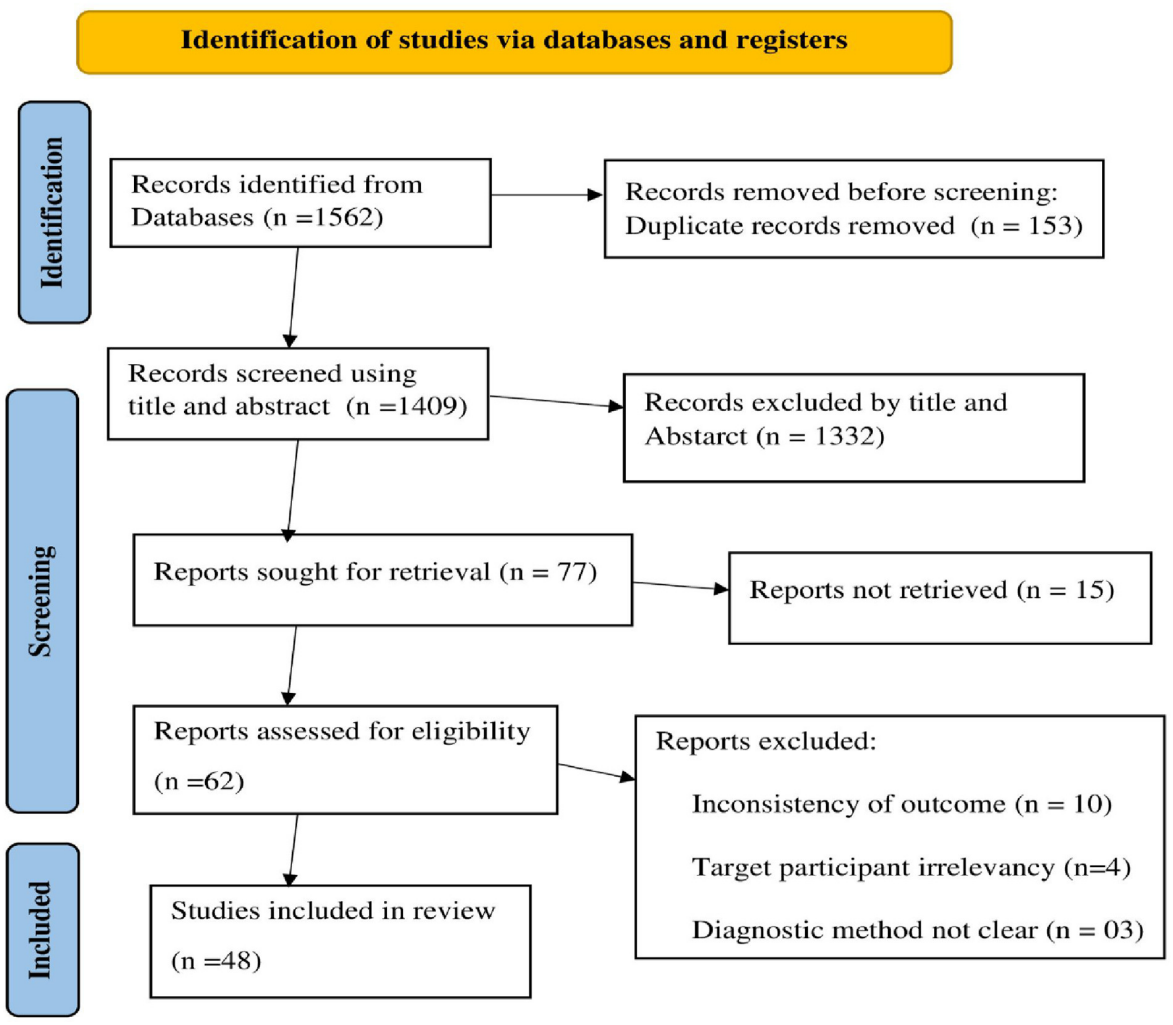
PRISMA flow diagram of the studies included in the systematic review and meta-analysis (32).

The reviewers independently collected the data from all accessible databases and records using a standardised form. During the data extraction, variables such as the author, year of publication, country, study subject, study design, sample population, number of cases (participants with the outcome), prevalence, contraceptive method type, and available predictor variables were captured and recorded using Microsoft Excel 2016 (Supplementary File S2). The overall process of study identification, screening, and selection process is presented using the 2020 updated PRISMA flow diagram (Figure 1).

### Measurement of the outcome

The first outcome variable of this study, the pooled prevalence of post-abortion contraceptive uptake in Africa, was calculated by dividing the total number of women who use modern contraceptives by the total number of women who received post-abortion services in the included studies (sample size) multiplied by 100. The second outcome variable of this study was the determinants of post-abortion contraceptive use in Africa, which were measured using an adjusted odds ratio. A separate met-analysis for each determinats of PAFP uptake was done using a subset of studies that reported adjusted odd ratios (AORs). A minimum of two studies was required for inclusion in each analysis. Data for each potential determinant factor was extracted in a two-by-two format in the Excel spreadsheet, and then the adjusted odds ratio was calculated for each factor. The potential determinant factors included in the analysis were residence (rural, urban), educational status (not attend formal education, attend formal education), marital status (in-union, not in-union), the desire of pregnancy (had pregnancy desire, had not pregnancy desire), knew of the fertile period (yes, no), post-abortion contraceptive counseling (yes, no), had a history of abortion (yes, no), trimester of abortion [first (<12 wks), second (>12 wks of gestation)], history of contraceptive use (yes, no), had contraceptive knowledge (yes, no), and gravidity (primigravida, multigravida).

### Data synthesis and statistical analysis

The data were imported into Stata version 15.1 from a Microsoft Excel sheet for analysis. The outcome variable, post-abortion contraceptive uptake, was calculated by dividing the number of women who use modern contraceptives by the total number of women who received post-abortion services, multiplied by 100. The pooled prevalence of post-abortion contraceptive uptake with a 95% confidence interval (95% CI) was determined using a random effects model.

The heterogeneity among the included studies was examined using *I*^2^ statistics. Heterogeneity was considered to exist when the *P*-value of *I*^2^ was less than 0.05. *I*^2^ statistics of 25, 50, and 75% represented low, moderate, and high heterogeneity, respectively (33). An Egger regression asymmetry test for meta-analysis was used to assess publication bias. A *P*-value <0.05 indicated the presence of publication bias.

Sub-group analysis and meta-regression were performed to verify the sources of heterogeneity. Additionally, a sensitivity analysis was carried out to ensure the robustness of the result and to check the influence of individual studies. To examine the influence of a single study, a pooled effect of PAC use with a 95% CI was calculated, leaving one study at a time. Meta-regression analysis was done to identify factors associated with the pooled effect of PAC uptake. The adjusted odds ratio with 95% CI was reported to show the strength of the association. Variables with a *P*-value <0.05 were considered statistically significant, highlighting their potential influence on PAC utilization.

## Results

Initially, a total of 1,562 articles were identified from different electronic databases, 153 of which were removed due to duplication. After screening the articles by title and abstract, 77 articles were selected for full-text retrieval. Fifteen additional articles were removed due to a lack of full-text articles or because they were published in languages other than English. Finally, of the 62 retrieved full-text articles, 48 articles were included in the analysis (Figure 1).

### Characteristics of the included studies

A total of 48 full-length articles published between January 1, 2015, and December 30, 2023, were included in this systematic review and meta-analysis. These articles originated from different countries, with a notable majority from Ethiopia (21 studies) and Ghana (6 studies). In terms of subregions, East Africa accounted for the highest number, with a total of 34 studies. The analysis encompassed data from 84,205 women who received postabortion care. On average, 1,754 women were included in each study, with sample sizes varying from 118 participants in Ethiopia to 29,056 in Ghana. Furthermore, 94% of the studies employed a cross-sectional design (Table 1).

**Table 1:** Characteristics of the 48 studies included in the systematic review and meta-analysis to estimate the pooled prevalence of postabortion contraceptive use in Africa.

S.no.	First author; year (reference)	Country	Study design	Sample size	No. of women use PAC
1	Belachew TB., et al; 2023 (34)	Ethiopia	Cross-sectional	1,236	316
2	Woldemichael D., et al; 2023 (35)	Ethiopia	Cross-sectional	272	146
3	Hinkosa L., et al; 2023 (36)	Ethiopia	Cross-sectional	221	164
4	Atuhairwe S., et al; 2023 (28)	Uganda	Cross-sectional	1,191	781
5	Sebazungu T., et al; 2023 (37)	Rwanda	Cross-sectional	250	92
6	Abebe BA., et al; 2022 (38)	Ethiopia	Cross-sectional	388	262
7	Mohammed U., et al; 2022 (39)	Ethiopia	Cross-sectional	471	348
8	Motuma VS., et al; 2022 (40)	Ethiopia	Cross-sectional	473	368
9	Atnafu E., et al; 2022 (41)	Ethiopia	cross-sectional	390	318
10	Tekle Lencha T., et al; 2022 (42)	Ethiopia	Cross-sectional	400	269
11	Baffour-Duah K., et al; 2022 (27)	South Africa	cross-sectional	12,006	8,941
12	Kalenga M., et al; 2022 (43)	Zambia	Cross-sectional	402	200
13	Fouelifack FY., et al; 2021 (44)	Cameroon	Cross-sectional	139	86
14	Muchie A., et al; 2021 (45)	Ethiopia	Cross-sectional	408	249
15	Teshome A., et al; 2021 (46)	Ethiopia	Cross-sectional	326	249
16	Agula C., et al; 2021 (47)	Ghana	Cross-sectional	251	47
17	Kayi EA., et al; 2021 (48)	Ghana	Cross-sectional	1,880	130
18	Wado YD., et al; 2021 (49)	Ethiopia	Cross-sectional	5,604	4,191
19	Satti I., et al; 2021 (50)	Sudan	Cross-sectional	1,077	223
20	Asubiojo B., et al; 2021 (51)	Tanzania	Cross-sectional	189	59
21	Malel ZJ., et al; 2021 (52)	South Sudan	Cross-sectional	285	22
22	Baynes C., et al; 2021 (16)	Tanzania	Cross-sectional	412	72
23	Atiglo DY., et al; 2020 (53)	Ghana	Cross-sectional	158	57
24	Salifu MG., et al; 2020 (54)	Ghana	Cross-sectional	3,039	1,130
25	Idris IM., et al; 2020 (55)	Eritrea	Cross-sectional	250	66
26	Madoué G., et al; 2020 (56)	Chad	Cross-sectional	135	81
27	Abate E., et al; 2020 (57)	Ethiopia	Cross-sectional	423	274
28	Millimouno TM., et al; 2019 (58)	Guinea	Cross-sectional	426	388
29	Pfitzer A., et al; 2019 (59)	Guinea	Cross-sectional	4,544	3,315
30	Abebe AM.,et al; 2019 (60)	Ethiopia	Cross-sectional	118	99
31	Mekuria A., et al; 2019 (61)	Ethiopia	Cross-sectional	400	314
32	Stephens B., et al; 2019 (62)	Tanzania	Cross-sectional	8,230	6,636
33	Mutua MM., et al; 2019 (15)	Kenya	Cross-sectional	2,568	1,424
34	Makenzius M.; 2018 (63)	Kenya	Prospective-follow-up	810	609
35	Hagos G., et al; 2018 (64)	Ethiopia	Cross-sectional	409	290
36	Moges Y., et al; 2018 (65)	Ethiopia	Cross-sectional	400	246
37	Asrat M., et al; 2018 (66)	Ethiopia	Cross-sectional	552	500
38	Agaba MN. 2018 (67)	Uganda	Cross-sectional	307	238
39	Kassahun M.; 2017 (68)	Ethiopia	Cross-sectional	459	316
40	Chukwumalu K., et al; 2017 (69)	Somalia	Cross-sectional	1,111	955
41	Abamecha A., et al; 2016 (70)	Ethiopia	Cross-sectional	399	241
42	Erko EK., et al; 2016 (71)	Ethiopia	Cross-sectional	184	129
43	Onyegbule OA., et al; 2016 (72)	Nigeria	Cross-sectional	480	383
44	Ibrahim WH., et al; 2015 (73)	Egypt	Quasi-experimental	276	77
45	Maxwell L., et al; 2015 (74)	Ghana	Cross-sectional	29,056	18,761
46	Rominski SD., et al; 2015 (75)	Ghana	Cross-sectional	612	341
47	Uwera J.; 2015 (76)	Kenya	Cross-sectional	174	106
48	Kokeb L., et al; 2015 (77)	Ethiopia	Cross-sectional	414	245

### Post-abortion contraceptive uptake in Africa

**Figure 2:**
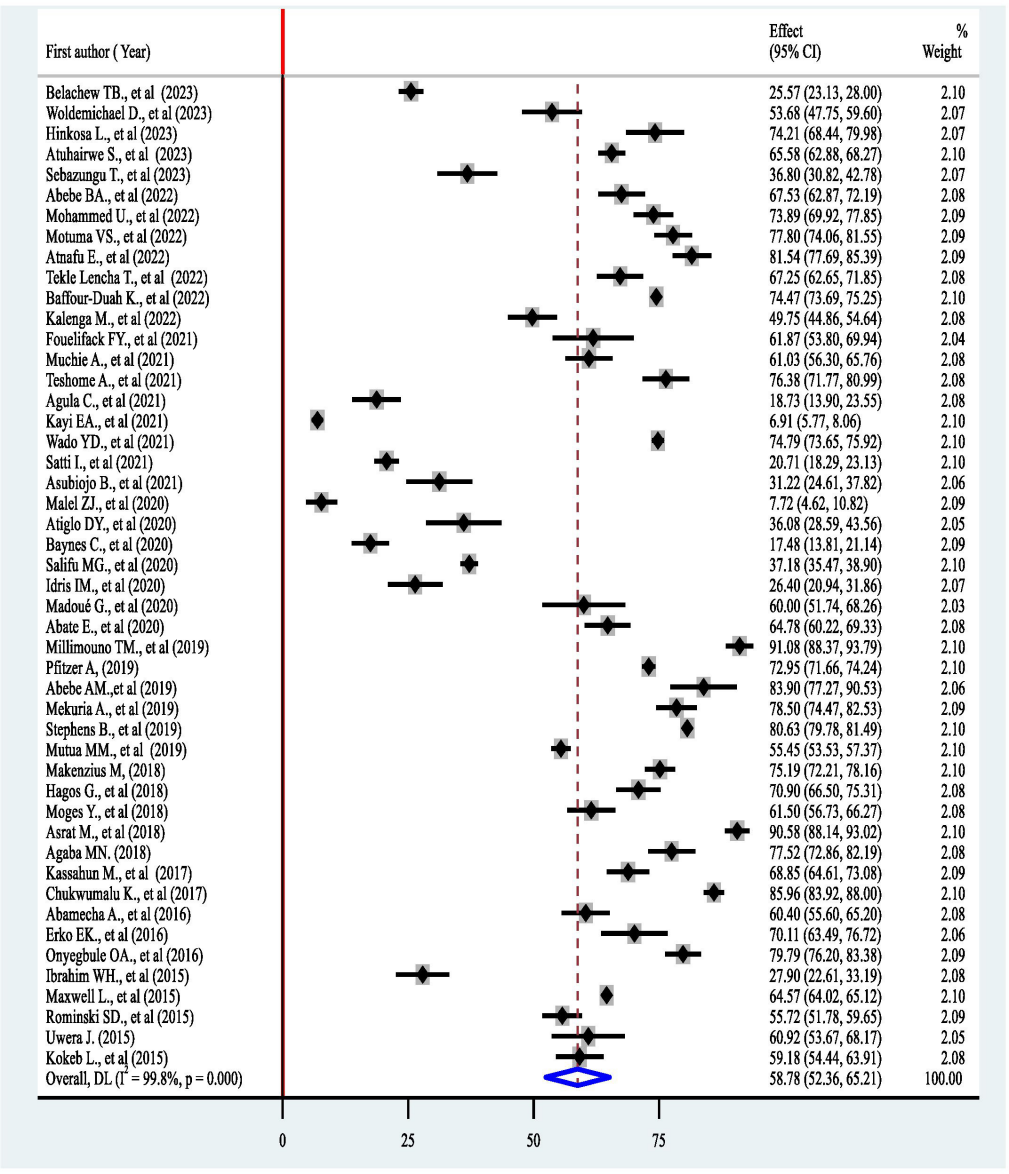
The pooled prevalence of postabortion contraceptive use in Africa.

In this systematic review encompassing 48 studies, the proportion of women using post-abortion contraceptives (PAC) varies significantly, with rates ranging from 6.92% in Ghana (48) to 91.08% in urban Guinea (58). The pooled prevalence of PAC uptake was found to be 58.78% (95% CI: 52.36–65.21). The *I*^2^ statistics indicated substantial heterogeneity (*I*^2^ = 99.8%, 95% CI: 99.2%–99.9%; *P* < 0.001) (Figure 2) Cochran's Q statistic was 20,014.24, with a *p*-value < 0.001, and the overall effect test yielded a *z* of 17.930 (*p* < 0.001). Consequently, subgroup analysis based on country, study design, sample size, and sub-region within Africa was performed to identify the sources of heterogeneity.

### Sub-group analysis

The subgroup analysis was performed using the following variables: country, sub-African regions, publication period, and sample size. According to the subgroup analysis by country, the highest prevalence of post-abortion contraceptive uptake (85.96%) was in Somalia, followed by 81.98% in Guinea, while the lowest was 7.72% in South Sudan, followed by 20.71% in Sudan. The country-level *I*^2^ statistics revealed heterogeneity between studies from the same countries (*p* < 0.001). On the other hand, the subgroup analysis of the Sub-African region showed the highest post-abortion contraceptive uptake in the southern African region (62.23%, 95% CI: 38.01–86.46) and the lowest in the West African region (51.48%, 95% CI: 32.35–70.61). However, a significant difference was not observed between the sub-African region. Regarding the sample size, heterogeneity was not observed among the three groups of samples: samples below or equal to 400, 400–1,000, or more than 1,000 (*p* = 0.250). However, there was heterogeneity within the groups in each sample (Table 2).

**Table 2:** Subgroup analysis of postabortion contraceptive use in Africa.

Subgroup	No. of included studies	Total sample size	Prevalence (95% CI)	Heterogeinity statistics		
				Cochran's *Q*	*I* ^2^	*P* value
By country				4,124.67		<0.001
Ethiopia	21	13,947	68.68 (61.27–76.08)	1,821.16	98.9%	<0.001
Uganda	02	1,498	71.39 (59.69–83.10)	18.86	94.7%	<0.001
Rwanda	01	250	36.80 (30.82–42.78)	–	–	–
South Africa	01	12,006	74.47 (73.69–75.25)	–	–	–
Zambia	01	402	49.75 (44.86–54.64)	–	–	–
Cameroon	01	139	61.87 (53.80–69.95)	–	–	–
Ghana	06	34,996	36.54 (9.74- 63.33)	8,431.67	99.9%	<0.001
Sudan	01	1,077	20.71 (18.29–23.13)	–	–	–
Tanzania	03	8,831	43.15 (4.76–91.06)	1,265.58	99.8%	<0.001
South Sudan	01	285	7.72 (4.62–10.82)	–	–	–
Eritrea	01	250	26.40 (20.94–31.86)	–	–	–
Chad	01	135	60.00 (51.74–68.26)	–	–	–
Guinea	02	4,970	81.98 (64.21–99.74)	–	–	–
Kenya	03	3,552	63.89 (49.24–78.55)	119.27	98.3%	<0.001
Somalia	01	1,111	85.96 (83.92–88.00)	–	–	–
Nigeria	01	480	79.79 (76.20–3.38)	–	–	–
Egypt	01	276	27.90 (22.61–33.19)	–	–	–
By Sub-African region				88.63		<0.001
East Africa	34	30,801	61.30 (53.93–68.67)	7,290.24	99.5%	<0.001
West Africa	09	40,446	51.48 (32.35–70.61)	10,162.00	99.9%	<0.001
North Africa	01	276	58.78 (52.36–65.21)	–	–	–
Central Africa	02	274	60.96 (55.18–66.73)	0.10	0.00	0.751
Southern Africa	02	12,408	62.23 (38.01–86.46)	95.82	99.9%	<0.001
By sample size				2.77		0.250
≤400	22	5,912	55.46 (44.42–66.50)	2,117.58	99.0%	<0.001
400–1,000	14	6,751	66.89 (56.33–77.45)	1,501.53	99.1%	<0.001
>1,000	12	71,542	55.41 (42.57–68.25)	15,825.54	99.9%	<0.001
Publication period				10.01		0.002
2015–2019	21	51,929	70.19 (65.28–75.10)	2,377.95	99.2%	<0.001
2020–2023	27	32,276	49.97 (38.44–61.49)	14,417.10	99.8%	<0.001

### Publication bias

**Figure 3:**
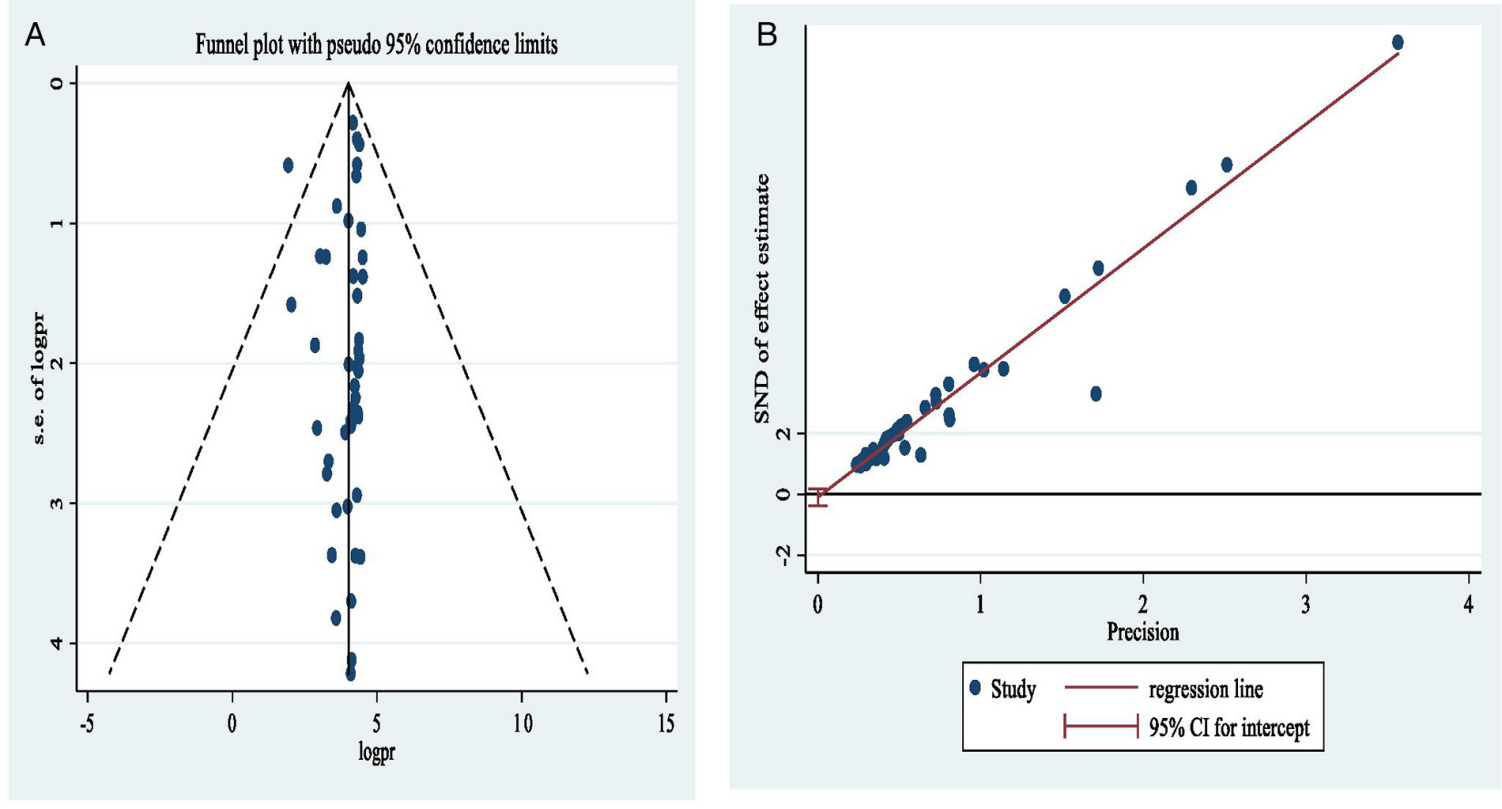
Funnel plot **(A)** and Egger's publication bias plot **(B)** of the logit event rate postabortion contraceptive uptake in Africa.

The funnel plot (Figure 3) and bias coefficient (Egger's test; *b* = −0.096, 95% CI = 0.372–0.180, *P* = 0.489) for studies published on contraceptive uptake among post-abortion women in Africa indicated no publication bias or small study effect present (Figure 3).

### Sensitivity analysis

To further investigate the sources of heterogeneity in the pooled prevalence of post-abortion contraceptive use in Africa and to ensure its robustness, we execute a sensitivity analysis. According to the sensitivity analysis result, post-abortion contraceptive uptake in Africa varied between 59.92% following the removal of Kayi EA., et al. (2021) (48) and 58.09% following the removal of Millimouno TM, et al. (2019) (58). Based on this analysis, the pooled prevalence of PAC uptake was robust and had no strong evidence that showed a single study influence (Table 3).

**Table 3:** Sensitivity analysis of pooled prevalence postabortion contraceptive use with a 95% in Africa for a single study was omitted.

Study omitted	Estimate	95% CI
Belachew TB., et al. (2023) (34)	59.5	53.10, 65.90
Woldemichael D., et al. (2023) (35)	58.89	52.39, 65.40
Hinkosa L., et al. (2023) (36)	58.46	51.96, 64.96
Atuhairwe S., et al. (2023) (28)	58.64	52.10, 65.18
Sebazungu T., et al. (2023) (37)	59.25	52.76, 65.74
Abebe BA., et al. (2022) (38)	58.6	52.10, 65.11
Mohammed U., et al. (2022) (39)	58.46	51.95, 64.97
Motuma VS., et al. (2022) (40)	58.38	51.87, 64.89
Atnafu E., et al. (2022) (41)	58.3	51.79, 64.80
Tekle Lencha T., et al. (2022) (42)	58.6	52.09, 65.11
Baffour-Duah K., et al. (2022) (27)	58.45	51.53, 65.36
Kalenga M., et al. (2022) (43)	58.98	52.47, 65.48
Fouelifack FY., et al. (2021) (44)	58.72	52.23, 65.22
Muchie A., et al. (2021) (45)	58.74	52.23, 65.25
Teshome A., et al. (2021) (46)	58.41	51.90, 64.92
Agula C., et al. (2021) (47)	59.63	53.18, 66.09
Kayi EA., et al. (2021) (48)	59.92	55.10, 64.74
Wado YD., et al. (2021) (49)	58.44	51.74, 65.14
Satti I., et al. (2021) (50)	59.6	53.24, 65.96
Asubiojo B., et al. (2021) (51)	59.36	52.88, 65.85
Malel ZJ., et al. (2020) (52)	59.88	53.55, 66.20
Atiglo DY., et al. (2020) (53)	59.26	52.77, 65.75
Baynes C., et al. (2020) (16)	59.67	53.24, 66.09
Salifu MG., et al. (2020) (54)	59.25	52.78, 65.71
Idris IM., et al. (2020) (55)	59.47	52.99, 65.95
Madoué G., et al. (2020) (56)	58.76	52.26, 65.26
Abate E., et al. (2020) (57)	58.66	52.14, 65.17
Millimouno TM., et al. (2019) (58)	58.09	51.62, 64.57
Pfitzer A, (2019) (59)	58.48	51.81, 65.15
Abebe AM.,et al (2019) (60)	58.26	51.76, 64.75
Mekuria A., et al. (2019) (61)	58.36	51.86, 64.87
Stephens B., et al. (2019) (62)	58.31	51.65, 64.98
Mutua MM., et al. (2019) (15)	58.85	52.27, 65.44
Makenzius M, (2018) (63)	58.43	51.91, 64.96
Hagos G., et al. (2018) (64)	58.53	52.01, 65.04
Moges Y., et al. (2018) (65)	58.73	52.23, 65.24
Asrat M., et al. (2018) (66)	58.1	51.63, 64.58
Agaba MN. (2018) (67)	58.39	51.88, 64.89
Kassahun M., et al. (2017) (68)	58.6	52.06, 65.08
Chukwumalu K., et al. (2017) (69)	58.2	51.70, 64.70
Abamecha A., et al. (2016) (70)	58.75	52.24, 65.26
Erko EK., et al. (2016) (71)	58.55	52.04, 65.05
Onyegbule OA., et al. (2016) (72)	58.33	51.83, 64.84
Ibrahim WH., et al. (2015) (73)	59.44	52.96, 65.92
Maxwell L., et al. (2015) (74)	58.66	51.15, 66.16
Rominski SD., et al. (2015) (75)	58.85	52.33, 65.36
Uwera J. (2015) (76)	58.74	52.24, 65.24
Kokeb L., et al. (2015) (77)	58.78	52.27, 65.28

### Types of post-abortion contraceptive use

Of a total of 48 studies, 28 (58.3%) examined the types of modern contraceptives that women received after undergoing an abortion. The pooled prevalence of post-abortion modern contraceptive uptake in these studies was 62.01%. Among the participants, 32, 705 (38.84%) received short-acting modern contraceptive methods, while 19,510 (23.17%) received long-acting modern contraceptive methods. Notably, more than half of the post-abortion contraceptive users received injectables, 16,892(20.06%) or implants, 14,113 (16.76%) (Table 4).

**Table 4:** Pooled prevalence of postabortion contraceptive use by method type in Africa.

Types of postabortion contraceptives	Pooled prevalence (95% CI)	I-squared
Sort-acting	38.84 (30.82, 46.86	99.8%, *P* < 0.001
Injectable	20.06 (11.37, 28.75)	99.8%, *P* < 0.001
Pills	11.72 (9.03, 14.41)	97.9%, *P* < 0.001
Condom	3.72 (1.24, 6.20)	97.5%, *P* < 0.001
Others	3.52 (0.37, 6.68)	98.5%, *P* < 0.001
Long-acting	23.17 (14.48, 31.86)	99.8%, *p* < 0.001
Implants	16.76 (9.9623.46)	99.7%, *P* < 0.001
Intra-Uterine Devices (IUDs)	6.44 (3.72, 9.16)	97.9%, *p* < 0.001
Sterilization	0.22 (−0.12, 0.56)	0.00%, *P* = 1.00

### Factors associated with post-abortion contraceptive uptake

The meta-analysis was performed on 29 of the included articles and included 11 identified potential predictor variables. The findings revealed that women who attended formal education (OR = 1.46, 95% CI = 1.03, 2.07), were married and/or in union (OR = 1.48, 95% CI = 1.01, 2.18), had awareness the period of fertility (OR = 1.72, 95% CI = 1.14, 2.59), were counseled about contraceptives (OR = 3.40, 95% CI = 1.82, 6.35), did not desire to become pregnant (OR = 3.08, 95% CI = 1.74, 5.44), had a history of contraceptive use (*R* = 4.35, 95% CI = 2.55, 7.42), and had contraceptive knowledge (OR = 2.30, 95% CI = 1.41, 3.76) had a statistically significant combined effect on post-abortion contraceptive uptake, with an overall effect of *P* < 0.05. However, residence, history of abortion, trimester of current abortion, and gravidity did not have significant combined effects on postabortion contraceptive use (Table 5).

**Table 5:** Factors associated with postabortion contraceptive use in Africa.

Variables	PAFP use		AOR (95% CI)	Overall effect (*P*-value)	Number of studies	References (*p* < 0.05)	References (*p* > 0.05)
	Yes, *n* (%)	No, *n* (%)					
Residence (*n* = 5,356)				0.209	07	(40, 41)	(37, 54, 55, 61, 66)
Rural	756	740	1.00				
Urban	2,032	1,828	1.44 (0.81, 2.55)				
Marital status (*n* = 10,067)				0.101	21	(37, 40–44, 51, 54, 60, 61, 63, 65, 66, 70, 71, 76, 77)	(38, 55, 67, 68)
In-union	3,390	2,364	1.48 (1.01, 2.18)				
Not in Union	2,445	1,868	1				
Attend formal education (*n* = 13,456)				0.033	14	(40, 45, 49, 65)	(39, 41, 43, 54, 55, 66, 68, 70, 76, 77)
Yes	6,913	4,035	1.46 (1.03, 2.07)				
No	1,753	894	1				
Knowing fertile period (*n* = 3,848)				0.010	03	(54, 64)	(61)
Yes	1,331	1,779	1.72 (1.14, 2.59)				
No	403	335	1				
Counseled about contraceptives (*n* = 6,627)				<0.001	16	(28, 35, 36, 38–40, 42, 43, 45, 60, 65, 68, 70, 73, 77)	(46)
Yes	3,028	1,265	3.40 (1.82, 6.35)				
No	1,213	1,121	1				
Have pregnancy desire (*n* = 1,951)				<0.001	05	(40, 41, 73)	(43, 61)
Yes	507	401	1				
No	755	288	3.08 (1.74, 5.44)				
Have abortion history (*n* = 5,191)				0.592	14	(35, 40–42, 45, 64, 68, 70)	(43, 44, 55, 61, 66, 67)
Yes	1,302	672	1.14 (0.71, 1.82)				
No	2,205	1,012	1				
Have a history of contraceptive use (*n* = 3,878)				<0.001	12	(36, 40–43, 60, 61, 65, 71)	(37, 51, 68)
Yes	1,800	618	4.35 (2.55, 7.42)				
No	732	728	1				
Gravidity (*n* = 7,990)				0.991	7	(41, 42, 49, 55, 63, 64)	(44)
Primi gravida	2,405	822	1.00 (0.61, 1.63)				
Multi Gravida	3,423	1,340	1				
Trimester of current abortion (*n* = 32,927)				0.263	8	(44, 49, 61, 74)	(38, 43, 51, 55)
1st (<12 weeks)	19,741	8,978	1.21 (0.87, 1.67)				
2nd (>12 weeks)	2,346	1,862	1				
Have contraceptive knowledge (*n* = 3,241)				0.001	9	(35, 36, 38, 41–43)	(39, 67, 68)
Yes	1,496	629	2.30 (1.41, 3.76)				
No	702	414	1				

## Discussion

Africa is a continent that has a high number of the least safe abortion service categories (78) and low accessibility and quality of family planning programs (79, 80). The provision of post-abortion contraceptive services is among the comprehensive abortion care packages that help to improve the health and well-being of women and reduce the repeated burden of abortion and related complications. To the best of the authors' knowledge, this study is the first to assess the predictors of postabortion contraceptives in Africa.

In this systematic review, 58.78% (95% CI: 52.36–65.21) of post-abortion contraceptive uptake was reported, which suggests that a potentially high impact could be expected on the risk of unintended pregnancy that will cause recurrent abortion. A similar finding was reported in a systematic review performed in Eastern Africa (67.86, 95% CI: 63.59, 72.12) (30) and Ethiopia (63.64%, 95% CI: 57.75–69.53) (29). However, the current finding was lower than that of a study performed in India (81%) (81), Nepal (79%) (82), the pooled prevalence of eight low-income countries (73%) (83), and 10 sub-Saharan and Asian countries (77%) (31). The possible reasons might be differences in the accessibility and quality of comprehensive post-abortion care services, women's knowledge of contraceptives, and women's desire for childbirth. The women's level of awareness of the fertile period, previous exposure to any contraceptive method, and current marital status might also contribute to the aforementioned differences.

According to our subgroup analysis, the lowest post-abortion contraceptive uptake was reported in South Sudan (7.7%) and Sudan (20.7%), and the highest was reported in Somalia (86%) and Guinea (82%). The reason for this great disparity in post-abortion contraceptive uptake between different countries could be the difference in national abortion and contraceptive strategies. The population culture, religion, population policy, and population stability of each country also have impacts on these differences.

According to the publication period, articles published before 2020 had a higher pooled prevalence of post-abortion contraceptive uptake (70.19%) than did articles published after 2020 (49.97%). A possible reason might be the influence of COVID-19. In the era of COVID-19, most health systems became fragile, and most routine health services were interrupted; the provision of contraceptive services was affected by the COVID-19 pandemic (84). One study revealed that at the time of the COVID-19 lockdown, women's access to contraception decreased from 70% to 60% (85). Moreover, the changing focus of the government health system and nongovernmental organizations in addressing the newly emerging and re-emerging diseases and other primary health care activities makes the provision of postabortion family planning services more challenging (86, 87).

Approximately two-thirds (64.10%, 95% CI: 53.56–74.65) of the post-abortion contraceptive acceptors used short-acting contraceptives. Among the specific contraceptive methods used, the most widely used contraceptive methods were injectables (30.27%, 95% CI: 18.96–41.57), followed by implants (25.13%, 95% CI: 16.75–33.51), oral contraceptive pills (22.34%, 95% CI: 16.76–27.92), IUDs (10.47%, 95% CI: 7.17–13.77), and male condoms (6.78%, 95% CI: 3.00–10.55%). Sterilization was the least used contraceptive method; only 0.34% of women used it. These findings are similar to those of studies performed in Nepal, East Africa, and developing countries (30, 82, 83). However, the current findings were different from those of a study performed in Atlanta, USA; 56% of post-abortion women used IUDs, implants, or sterilization (88). In a study done in China, approximately half of the participants used long-acting post-abortion contraceptives, and the three most utilized types of contraceptives were condoms (41.3%), IUDs (40.3%), and sterilization (9.2%) (89). This difference might be due to differences in participants' knowledge of effective and safe contraceptives, the quality of counseling, or the availability of different contraceptive methods. Most women in developing countries, including Africa, face many challenges in using the most preferred contraceptive method; inadequate trained providers, limited contraceptive method mix availability, and stockouts are among them (86, 90). In addition, participants' myths and misconceptions about each contraceptive method type and provider attitude and skill in delivering the service would have an impact on the aforementioned differences.

According to the results of this systematic review and meta-analysis, women who attended formal education had greater odds of using post-partum contraceptives than women who did not. These findings were supported by other studies (91–94). This might be because education has the power to increase women's contraceptive knowledge and attitudes (95). On the other hand, contraceptive knowledge and attitudes have an impact on the use of postabortion contraceptives. In this study, women who had contraceptive knowledge had higher odds of using post-abortion contraceptives. Other previous studies performed in the USA, China, and Singapore also supported these findings (96–98).

Like in other previous studies (29, 30, 99–101), the pooled odds ratio of post-abortion contraceptive uptake was greater among women who received post-abortion contraceptive counseling than among those who did not receive it. This is because counseling has the power to change the behavior of individuals by addressing the unique perceptions and attitudes of women through the use of person-centered, well-structured counseling approaches (100, 102, 103). Postabortion contraceptive counseling can enable women to make informed decisions about their fertility. In addition, counseling can enable women to understand the period of fertility (a period at risk for conception) (104). In this study, women who were aware of the fertility period had higher pooled odds of using postabortion contraceptives than women who were not aware of this period.

In this study, a history of contraceptive use was positively associated with post-abortion contraceptive use. These findings are supported by previous studies performed in Eastern Africa, Pakistan, and Nepal, as well as a systematic review performed in Ethiopia (29, 30, 101, 105). As a result of being exposed to family planning services, women would develop a positive lived experience with different contraceptives, and they are less affected by different contraceptive myths and misconceptions. A study in Nigeria revealed that women with lower misconception scores were significantly more likely to use modern contraceptives (106, 107). Moreover, women who have a history of contraceptive use have better information about the service than women who do not; this might have a positive effect on the use of current postabortion contraceptives. A study performed in Colombia reported a history of contraceptive use as an enabler of current contraceptive use (108).

The desire for pregnancy is another important variable that is significantly associated with postabortion contraceptive uptake; the pooled odds ratio of post-abortion contraceptive uptake was greater among women who did not desire pregnancy than among their counterparts. This was similar to the findings of a study performed in China (98). However, in a study performed in Brazil, planning of pregnancy and fertility desire were not significantly associated with postabortion contraceptive uptake (104).

## Limitations of the study

This systematic review and meta-analysis have some limitations; the study didn't report the pooled prevalence of post-abortion contraceptive uptake among women who had a demand for contraceptives separately due to the unavailability of clearly stated data from the included studies. Additionally, many of the included studies were cross-sectional, which restricts the ability to infer cause-and-effect relationships due to the simultaneous measurement of exposure and outcome. While the application of the random effect model is appropriate for accounting for study variability, this approach can lead to wide confidence intervals and may not fully address the underlying sources of heterogeneity. Consequently, the pooled prevalence estimate should be interpreted considering these methodological constraints.

## Conclusion

Post-abortion contraceptive uptake in Africa is low; among five women, two leave the facility without modern contraceptives. Even this figure reaches 50% or more in studies published after 2020. The main predictors identified for post-abortion contraceptive uptake were attending formal education, marital status, knowledge of the fertility period, post-abortion contraceptive counseling, history of contraceptive use, desire for pregnancy, and contraceptive knowledge. Therefore, efforts should be made to strengthen post-abortion counseling, women's education, and awareness creation about the period of fertility and contraceptives. Moreover, strengthening awareness creation programs for post-abortion women to postpone the desire for pregnancy at least six months after termination of pregnancy is paramount to reduce the cycling of abortion. These findings might also provide useful information for policymakers and programmers in developing policy briefs and evidence-based intervention modalities to improve post-abortion contraceptive use in low-income countries.

## Data Availability

The original contributions presented in the study are included in the article/Supplementary Material, further inquiries can be directed to the corresponding author.

## Supplementary material

Supplementary File S1 Searching strategy.docx.

Supplementary File S2 Data base.xlsx.

Supplementary File S3 PRISMA_2020_checklist.docx.

Supplementary File S4 Forest plot of the subgroup analysis.docx.

Supplementary File S5 Forest-polt of factors for PAC use in Africa.docx.
