# Supplementary material for: Post-abortion contraceptive uptake, choices, and factors associated with it among women seeking abortion services in Africa: a systematic review and meta-analysis
Source: Front Glob Womens Health. 2025 Jun 16;6:1478797. doi: 10.3389/fgwh.2025.1478797 (PMC12206890; doi:10.3389/fgwh.2025.1478797)
Supplement: Supplementary File S1 — Searching strategy.docx. [file Table1.docx]

| Search | Query | Items retrieved |
| --- | --- | --- |
| PubMed/Medline | ((("Abortion, Induced"[Mesh]) AND ("Contraceptive Agents"[Mesh])) OR ("Family Planning Services"[Mesh])) AND ("Africa"[Mesh]) | 2800 |
| From 2015/1/1 - 2023/12/31 |  | 1106 |
| PubMed/Medline/NIH using All fields | (post[All Fields] AND ("abortion, induced"[MeSH Terms] OR ("abortion"[All Fields] AND "induced"[All Fields]) OR "induced abortion"[All Fields] OR "abortion"[All Fields]) AND ("contraceptive agents"[All Fields] OR "contraceptive devices"[MeSH Terms] OR ("contraceptive"[All Fields] AND "devices"[All Fields]) OR "contraceptive devices"[All Fields] OR "contraceptive"[All Fields] OR "contraceptive agents"[MeSH Terms] OR ("contraceptive"[All Fields] AND "agents"[All Fields]) OR "contraceptive agents"[All Fields]) AND ("africa"[MeSH Terms] OR "africa"[All Fields])) AND ("open access"[filter] AND ("2015/01/01"[PubDate] : "2023/12/31"[PubDate])) | 2102 |
| AJOL | post "abortion" "contraceptive" use and associated factors in Africa from 2015 to December 2023 | 46 |

**^S1 File : Searching strategy^**
