# Supplementary material for: Post-abortion contraceptive uptake, choices, and factors associated with it among women seeking abortion services in Africa: a systematic review and meta-analysis
Source: Front Glob Womens Health. 2025 Jun 16;6:1478797. doi: 10.3389/fgwh.2025.1478797 (PMC12206890; doi:10.3389/fgwh.2025.1478797)
Supplement: Supplementary File S4 — Forest plot of the subgroup analysis.docx. [file Table4.docx]

Figure 1. Forest plot of the sub-group analysis pooled prevalence of postabortion contraceptive use by country in Africa

Figure 2. Forest plot of the sub-group analysis pooled prevalence of postabortion contraceptive use by publication period in Africa

Figure 3. Forest plot of the sub-group analysis pooled prevalence of postabortion contraceptive use by sub-African region.

Figure 4. Forest plot of the sub-group analysis pooled prevalence of postabortion contraceptive use by number of sample size in Africa
