# Supplementary material for: Post-abortion contraceptive uptake, choices, and factors associated with it among women seeking abortion services in Africa: a systematic review and meta-analysis
Source: Front Glob Womens Health. 2025 Jun 16;6:1478797. doi: 10.3389/fgwh.2025.1478797 (PMC12206890; doi:10.3389/fgwh.2025.1478797)
Supplement: Supplementary File S5 — Forest-polt of factors for PAC use in Africa.docx. [file Table5.docx]

Figure 1. The overall pooled odds ratio of the association between residence and post-abortion contraceptive use in Africa.

Figure 2. The overall pooled odds ratio of the association between marital status and post-abortion contraceptive use in Africa.

Figure 3. The overall pooled odds ratio of the association between marital status and post-abortion contraceptive use in Africa.

Figure 4. The overall pooled odds ratio of the association between knowledge of fertile period and post-abortion contraceptive use in Africa.

Figure 5. The overall pooled odds ratio of the association between post-abortion contraceptive counseling and post-abortion contraceptive use in Africa.

Figure 6. The overall pooled odds ratio of the association between desire of pregnancy and post-abortion contraceptive use in Africa.

Figure 7. The overall pooled odds ratio of the association between history of abortion and post-abortion contraceptive use in Africa.

Figure 8. The overall pooled odds ratio of the association between history of contraceptive use and post-abortion contraceptive use in Africa.

Figure 9. The overall pooled odds ratio of the association between trimester of abortion and post-abortion contraceptive use in Africa.

Figure 10. The overall pooled odds ratio of the association between gravidity and post-abortion contraceptive use in Africa.

Figure 11. The overall pooled odds ratio of the association between gravidity and post-abortion contraceptive use in Africa.
